# Chronic Exposure to Flavored Tobacco Aerosols Induces Inflammation and Hepatic Mitochondrial Impairment in Pregnant Versus Non-Pregnant Mouse Cohorts

**DOI:** 10.3390/ijms27219851

**Published:** 2026-11-03

**Authors:** Kamryn Sample, Manjula M. Venkatappa, Young Do Koo, Zachary Foulks, Vingie Ng, Morgan Shaffer, Mei L. Lietsch, Andrea Adamcakova-Dodd, Peter S. Thorne, Kyle S. McCommis, Devaraja Sannaningaiah, Rose M. Robertson, Vernat Exil

**Affiliations:** 1 Department of Pediatrics, Division of Pediatric Cardiology, Saint Louis University, St. Louis, MO 63104, USA; kamryn.sample@slu.edu (K.S.); mvmanju134@gmail.com (M.M.V.); 2 Department of Medicine, David Geffen School of Medicine, UCLA, Los Angeles, CA 90095, USA; ykoo@mednet.ucla.edu; 3 Department of Biochemistry, Saint Louis University, St. Louis, MO 63104, USA; zachary.foulks@health.slu.edu (Z.F.); kyle.mccommis@health.slu.edu (K.S.M.); 4 Department of Occupational and Environmental Health, University of Iowa, Iowa City, IA 52242, USA; vingie-ng@uiowa.edu (V.N.); andrea-a-dodd@uiowa.edu (A.A.-D.); peter-thorne@uiowa.edu (P.S.T.); 5 Department of Pediatrics, Division of Pediatric Cardiology, Carver College of Medicine, Iowa City, IA 52242, USA; morgan-a-shaffer@uiowa.edu (M.S.); mei-lietsch@uiowa.edu (M.L.L.); 6 Department of Studies and Research in Biochemistry, Tumkur University, Tumkur 572118, Karnataka, India; sdevbiochem@gmail.com; 7 Department of Medicine, Vanderbilt University Medical Center, Nashville 37232, TN, USA; rosemarie.robertson@heart.org; 8 American Heart Association, Dallas, TX 75231, USA

**Keywords:** electronic cigarettes(eCig), pulmonary inflammation, mitochondrial dysfunction, virginia tobacco, menthol, cytokines

## Abstract

This study was conducted to investigate the impact of Virginia tobacco (VT), Menthol (MT), and sham aerosols on both normal and pathological responses in pregnant and non-pregnant mouse groups. Aerosol vaping exposure and body weight analysis were evaluated in pregnant and non-pregnant cohorts and compared with controls exposed to sham aerosols in each group. We assessed pulmonary inflammation using the Bronchoalveolar Lavage Fluid (BALF) total cell count, total protein, and lactate dehydrogenase (LDH) activity. In addition, we measured hepatic triglyceride levels and lipogenic genes in the liver, such as Scd1, Fasn, Srebp1c1, and the estrogen receptor (Esr1), via RT-qPCR and Western blot analysis. Liver fibrosis was assessed via histopathology and mitochondrial functional assays, and serum cytokines were measured in each group. In pregnant mice exposed to VT, pulmonary inflammation, increased cell counts and protein content, and no change in lactate dehydrogenase activity in Bronchoalveolar Lavage Fluid (BALF) were observed compared with non-pregnant mice. VT exposure appeared to cause direct adverse health outcomes leading to increased expression of liver lipogenic genes, such as *Scd1*, *Fasn*, *Srebp1c*, and estrogen receptors. Maternal liver lipid equilibrium was disrupted upon VT exposure. Vaping either VT or MT significantly reduced mitochondrial oxidative function in the livers of pregnant mice but not in those of non-pregnant mice. This study’s findings suggest that pregnancy may be associated with distinct immunological responses to VT and MT exposure. VT exposure was associated with altered hepatic lipogenic gene expression, suggesting potential metabolic effects on the maternal liver, whereas MT exposure was associated with liver structural changes and fibrosis-related outcomes.

## 1. Introduction

Over the past two decades, the use of electronic nicotine-delivery systems (ENDSs) and electronic cigarettes (eCigs) has increased exponentially across the globe. An important motivation for vaping is the public perception that it is completely harmless or a safer alternative to smoking combustible tobacco. Multiple studies have demonstrated that the aerosol produced by eCigs is not just water vapor [1]; it includes carrier solvents (propylene glycol (PG) and vegetable glycerine (VG), nicotine (whether natural or synthetic), flavoring chemicals, various heavy metals such as nickel, tin, lead, chromium and/or cadmium, and differing amounts of other chemicals (formaldehyde, acetaldehyde, acrolein, benzene, toluene, xylenes, styrene, nitrosamines, and silicon conjugates) [2]. As a result, eCig use may induce addiction to nicotine and a variety of adverse biological and health-related outcomes, such as oxidative stress, inflammation, tissue damage, hormonal imbalance and neuronal dysfunction [3]. Previous reports suggest that adverse effects on the respiratory system, risk factors for cardiovascular disease, potential harm to brain development, and poor sleep quality are associated with eCig use. Additionally, researchers have shown that cigarette smokers and eCig users show similar as well as distinct patterns of gene dysregulation [4].

Lungs are the main port of entry for eCig aerosols; therefore, they have the greatest exposure to the multiple harmful or potentially harmful constituents (HPHCs) of these aerosols and may be at higher risk of vaping-induced pathological changes [5]. These chemical components have been shown to disrupt normal respiratory tract physiology by activating inflammatory cascades. The pathophysiological consequences of e-cigarettes with specific flavors, such as Virginia Tobacco (VT), have not been sufficiently investigated, especially during pregnancy, despite prior research demonstrating the health effects and toxicities of numerous e-cigarettes.

Pregnancy causes intense immunological, metabolic, and hormonal changes to protect the developing fetus and maintain maternal health. Smoking cigarettes during pregnancy negatively affects fetal brain development [6]. During the gestational period, metabolic reprogramming of the maternal liver often alters lipogenic pathways to ensure that nutrients are delivered to the fetal placental unit. In addition, systemic immune responses may shift toward a subtly different pro- and anti-inflammatory balance [7]. The mechanism by which pregnancy-specific variations respond to xenobiotic stress from eCig aerosols remains unknown. However, studies have demonstrated the teratogenicity of nicotine and the way in which the maternal system copes with vaping-induced stress in comparison to non-pregnant cohorts. Specifically, comparisons of VT and MT aerosols’ effects on pulmonary inflammation, hepatic de novo lipogenesis, and mitochondrial function in pregnant and non-pregnant women have not been previously evaluated. In this study, Virginia Tobacco (VT)- and Menthol (MT)-flavored JUUL (JUUL Labs, San Francisco, CA) products were investigated to determine if variations in taste formulations affect inflammatory reactions and hepatic mitochondrial function after prolonged exposure to tobacco aerosols. The aim was to determine whether the biological consequences of exposure differ depending on the type of flavor by comparing these two products and to better understand the role of pregnancy-specific factors, like the fetal placental unit or changes in hormonal profile, in aggravating or alleviating vaping-induced toxicity. The findings will be crucial for developing clinical guidelines and targeted public health interventions. This article reports the effect of VT and MT aerosol exposure on pregnant and non-pregnant mice models. The results were obtained by evaluating pulmonary inflammatory markers in bronchopleural lavage fluid (BALF) and characterizing lipogenic genes in the liver, such as Scd1, Fasn, and Srebp1c and estrogen receptors, along with the dynamics of systemic cytokines and mitochondrial oxidative function.

## 2. Results

### 2.1. Aerosol Exposure Affects Variation in Gestational Weight

**Figure 1:**
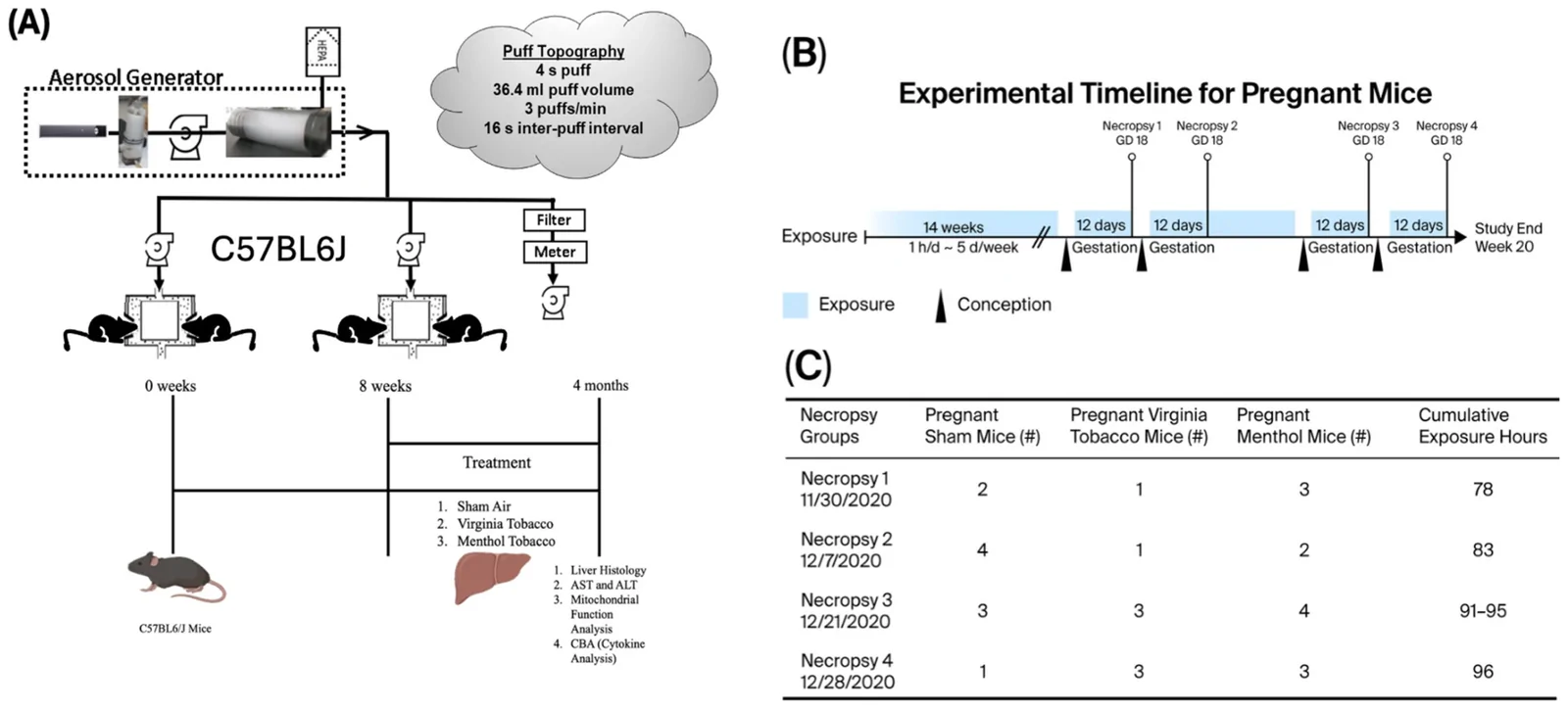
Experimental design and exposure setup. (**A**) Schematic of nose-only inhalation system (aerosol generator, puff topography: 36.4 mL/puff, 3 puffs/min, 16 s inter-puff interval) using C57BL6J mice. (**B**) Experimental timeline for pregnant mice. (**C**) Necropsy timeline for pregnant animals. (The picture of mice and liver illustration were created in BioRender: Sample, K. (2026) https://BioRender.com/2u6gayt and Sample, K. (2026) https://BioRender.com/09pre2m).

We studied vaping using puff-topography parameter analysis with sham, Virginia tobacco (VT), and Menthol (MT) aerosols, attributing downstream biological differences to aerosol constituents rather than exposure mechanics (Figure 1A). We studied CS7BL/6J females for 13 weeks, with pre-mating exposure followed by continued exposure through 12 days of gestation. The pregnant group of mice (Figure 1B) were chronically exposed to aerosols before conception and throughout gestation, with a fixed necropsy timeline in each mouse to standardize the gestational timing. Figure 1C shows that the pregnant mice were euthanized in 4 cohorts (at 78 h, 83 h, 91–95 h, and 96 h).

**Figure 2:**
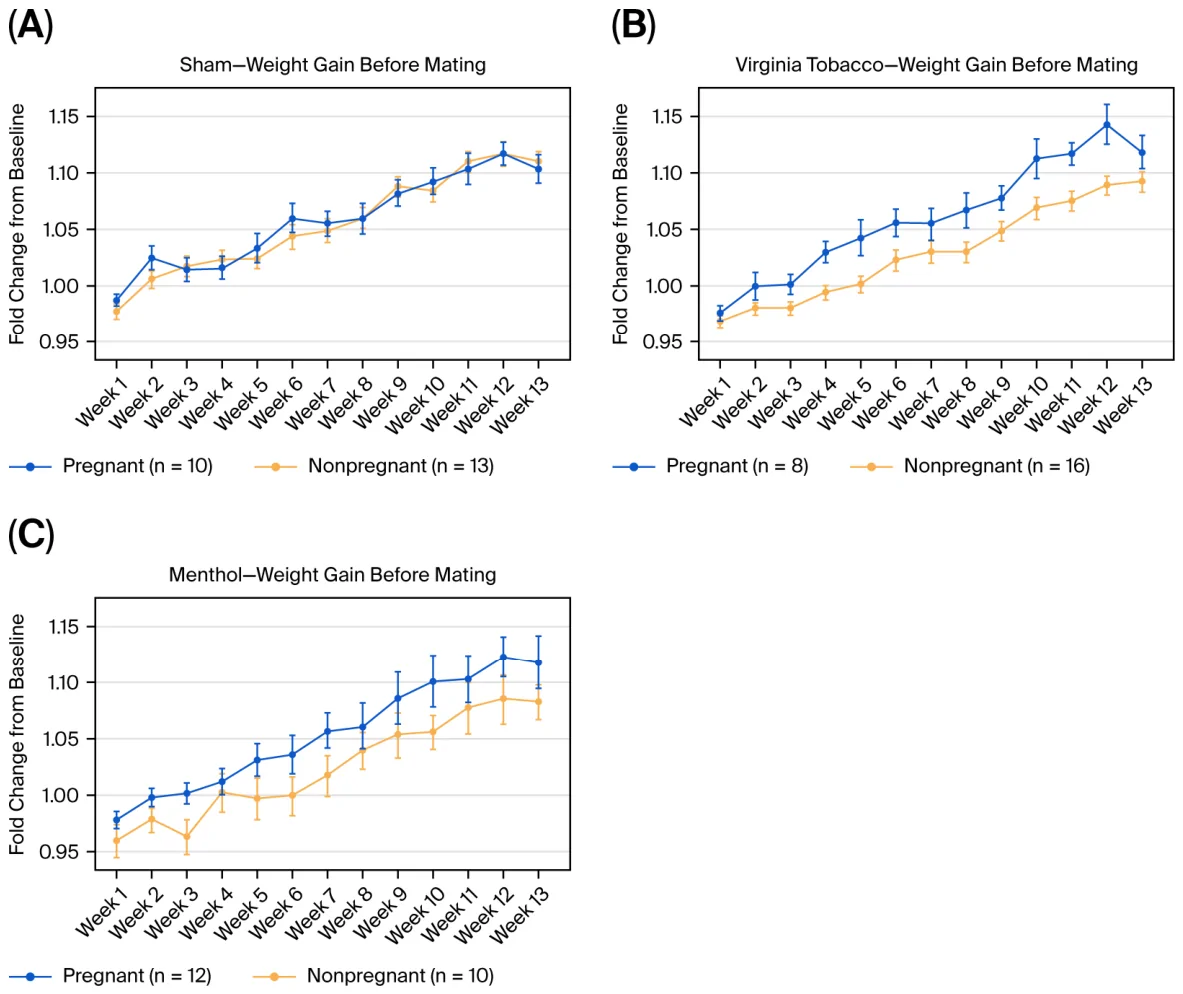
Body weight fold change analysis in pregnant and non-pregnant females. (**A**) Sham group fold change analysis. (**B**) Virginia tobacco group fold change analysis. (**C**) Menthol group fold change analysis.

Body weight was monitored for 13 weeks before mating in the sham, Virginia tobacco (VT), and menthol tobacco (MT) groups. Animals that subsequently became pregnant generally exhibited greater pre-mating body weight gain than those that remained non-pregnant, with more pronounced differences observed in the VT- and MT-exposed groups (Figure 2A–C).

### 2.2. VT Induces Vascular Permeability in Pregnant Cohorts While MT Elicits Pro-Inflammatory Infiltration in Non-Pregnant Cohorts

**Figure 3:**
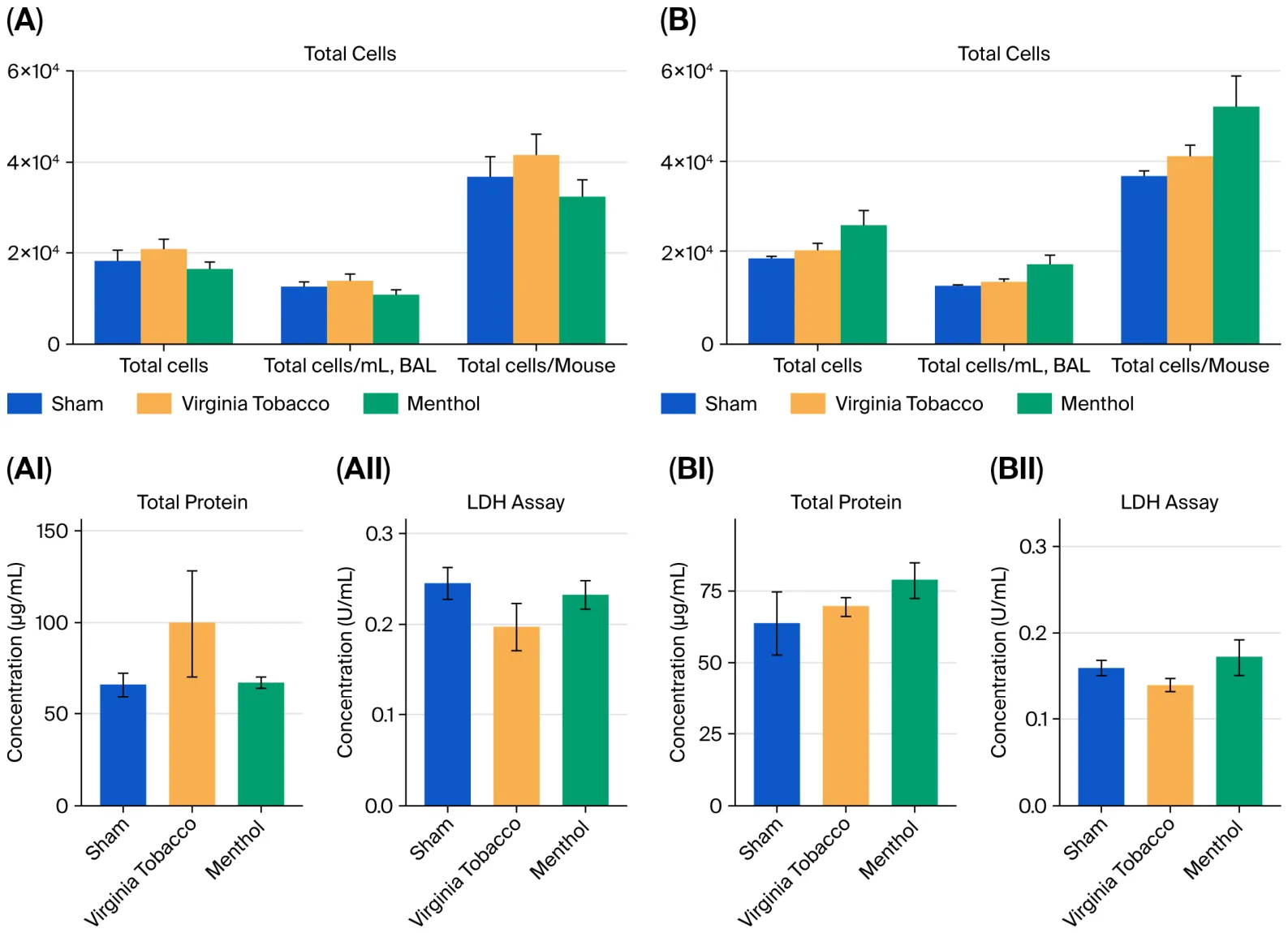
Bronchoalveolar lavage (BAL) analysis. (**A**) Chronic exposure of pregnant cohort: BAL analysis and total cell count. (**AI**) Total protein analysis in pregnant cohort. (**AII**) LDH analysis in pregnant cohort. (**B**) Chronic exposure of non-pregnant cohort: BAL analysis and total cell count. (**BI**) Total protein analysis in non-pregnant cohort. (**BII**) LDH analysis in non-pregnant cohort.

All groups underwent bronchoalveolar lavage (BAL) to measure pulmonary inflammation in treated pregnant and non-pregnant animals. Figure 3A shows the treated pregnant group, with increased BAL cell counts after vaping exposure. In the VT-treated pregnant group, total cells, total BAL cell counts, and total cells per mouse were slightly elevated compared to the sham and MT groups. To confirm BAL expression, we analyzed LDH and total protein (Figure 3AI). The total protein concentration was higher in VT-exposed animals than in the sham and MT groups. However, the LDH concentration was slightly lower in the VT-exposed animals (Figure 3AII). Figure 3B shows the total cellular analysis of treated non-pregnant animals, suggesting that, compared with the VT and sham animals, MT-exposed animals showed elevated total cells, total BAL cell counts, and total cells per mouse. Figure 3BI depicts the total protein content in the MT-treated group and increased levels of LDH (Figure 3BII) compared to the VT and sham-group animals. This observation suggests that the VT- and MT-induced inflammation observed in the pregnant and non-pregnant animals reflects the engagement of different pathways. Enhanced cell counts in the MT aerosol-exposed non-pregnant mice showed a pro-inflammatory effect on lung tissue. While VT exposure in the pregnant cohort showed cellular infiltration, pregnancy-related hormonal changes may dampen the response to MT.

### 2.3. Menthol Aerosol Exposure Alters Hepatic Triglycerides and Lipogenic Gene Expression in Pregnant vs. Non-Pregnant Mice

**Figure 4:**
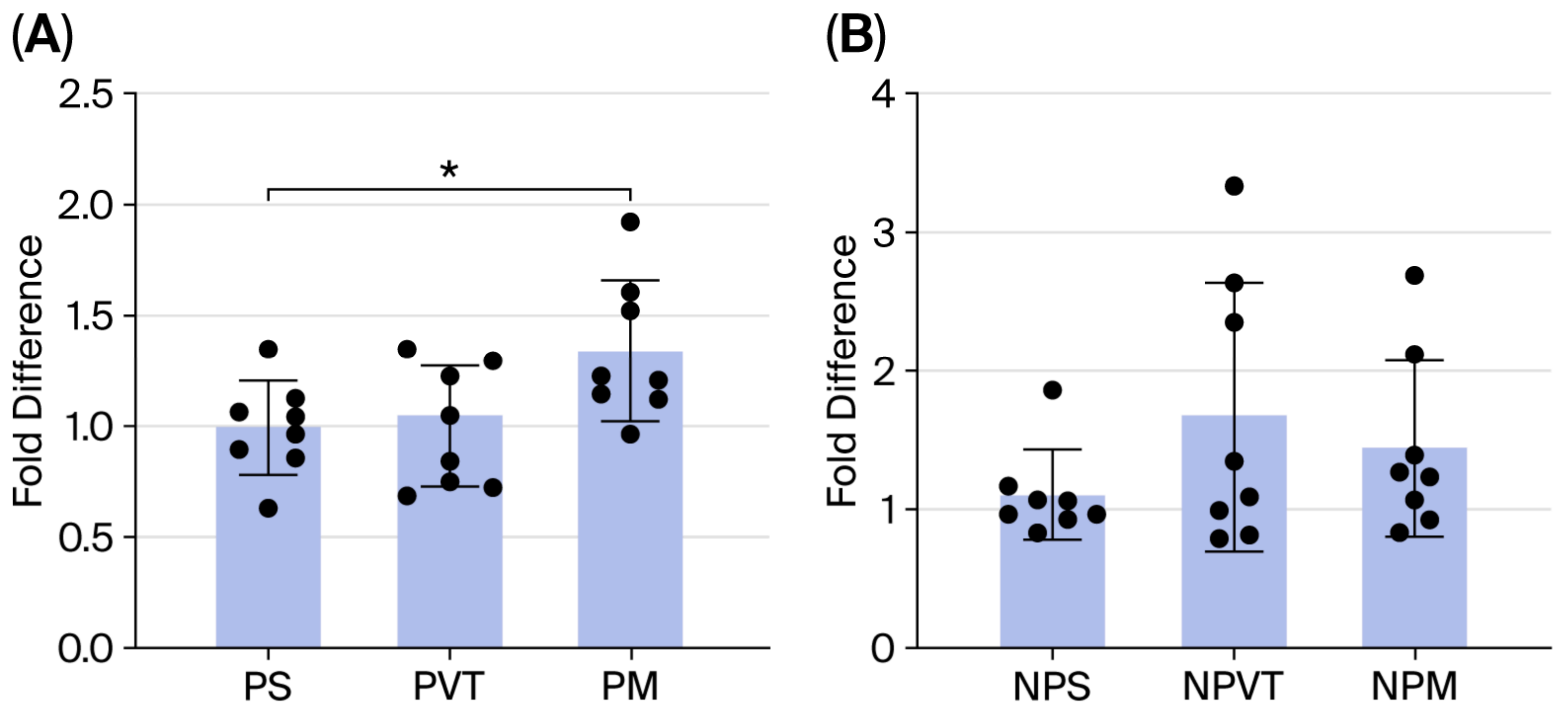
Triglyceride analysis in pregnant and non-pregnant cohorts. (**A**) Fold of difference in triglycerides in pregnant cohorts. (**B**) Fold of difference in triglycerides in non-pregnant cohorts. PS = Pregnant Sham; PVT = Pregnant Virginia Tobacco; PM = Pregnant Menthol; NPS = Non-Pregnant Sham; NPVT = Non-Pregnant Virginia Tobacco; NPM = Non-Pregnant Menthol.

**Figure 5:**
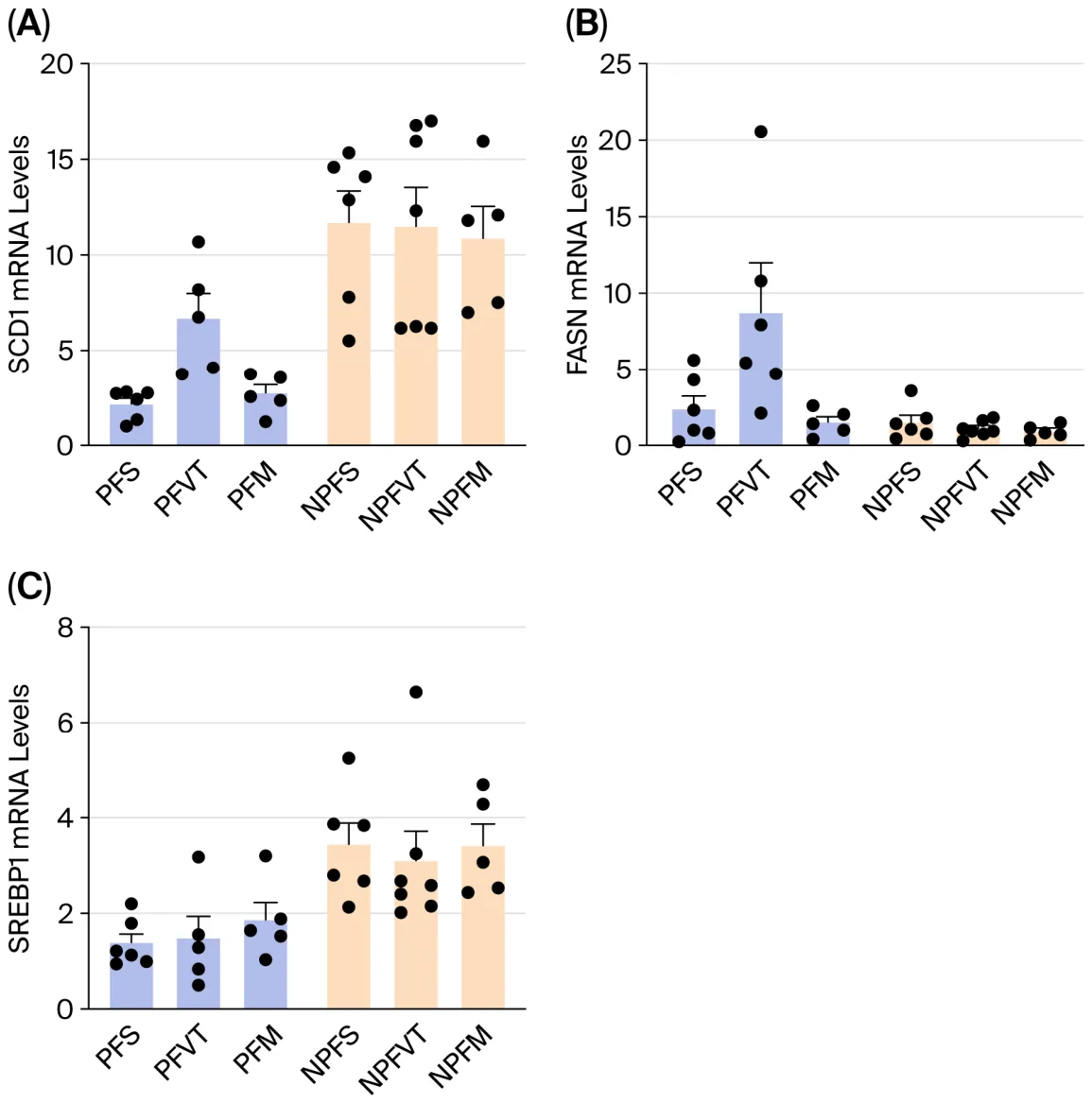
mRNA level analysis in pregnant and non-pregnant cohorts: (**A**) SCD1 mRNA level analysis; (**B**) FASN mRNA level analysis; and (**C**) SREBP1 mRNA level analysis. PFS = Pregnant Sham; PFVT = Pregnant Virginia Tobacco; PFM = Pregnant Menthol; NFPS = Non-Pregnant Sham; NFPVT = Non-Pregnant Virginia Tobacco; NFPM = Non-Pregnant Menthol.

We analyzed hepatic triglyceride levels in both the treated pregnant and non-pregnant cohorts. Figure 4A represents the fold change in triglycerides in the pregnant group, showing that compared to the sham and VT groups, the MT-treated cohorts exhibited the highest fold difference in liver triglycerides; however, the differences between the sham and VT groups did not reach statistical significance. Figure 4B indicates that the non-pregnant animals exhibited a trend toward elevated fold differences in triglycerides in the VT groups. The occurrence of menthol-specific triglyceride accumulation in treated pregnant animals suggests that pregnancy may substantially potentiate the hepatotoxic lipid-accumulating effect of menthol aerosol exposure. Furthermore, hepatic mRNA levels were measured to demonstrate the metabolic pathway with key markers such as *Scd1*, *Fasn*, and *Srebp1c*. In the VT-treated pregnant group, the mRNA levels for *Scd1* and *Fasn* (Figure 5A,B) were upregulated compared to in the sham and MT groups. Interestingly, livers from non-pregnant females showed higher baseline *Scd1* mRNA levels in all treatment groups (Figure 5A). This was not observed for *Fasn* expression (Figure 5B); however, *Srebp1c* was also slightly elevated in the livers of non-pregnant compared to pregnant mice (Figure 5C). This suggests that pregnancy significantly influences hepatic lipid metabolism, and VT exposure can alter this metabolic reorganization in pregnant mice.

### 2.4. Downregulation of SREBP1 and Alteration of Estrogen Receptor a Protein Levels Are Influenced by Pregnancy

**Figure 6:**
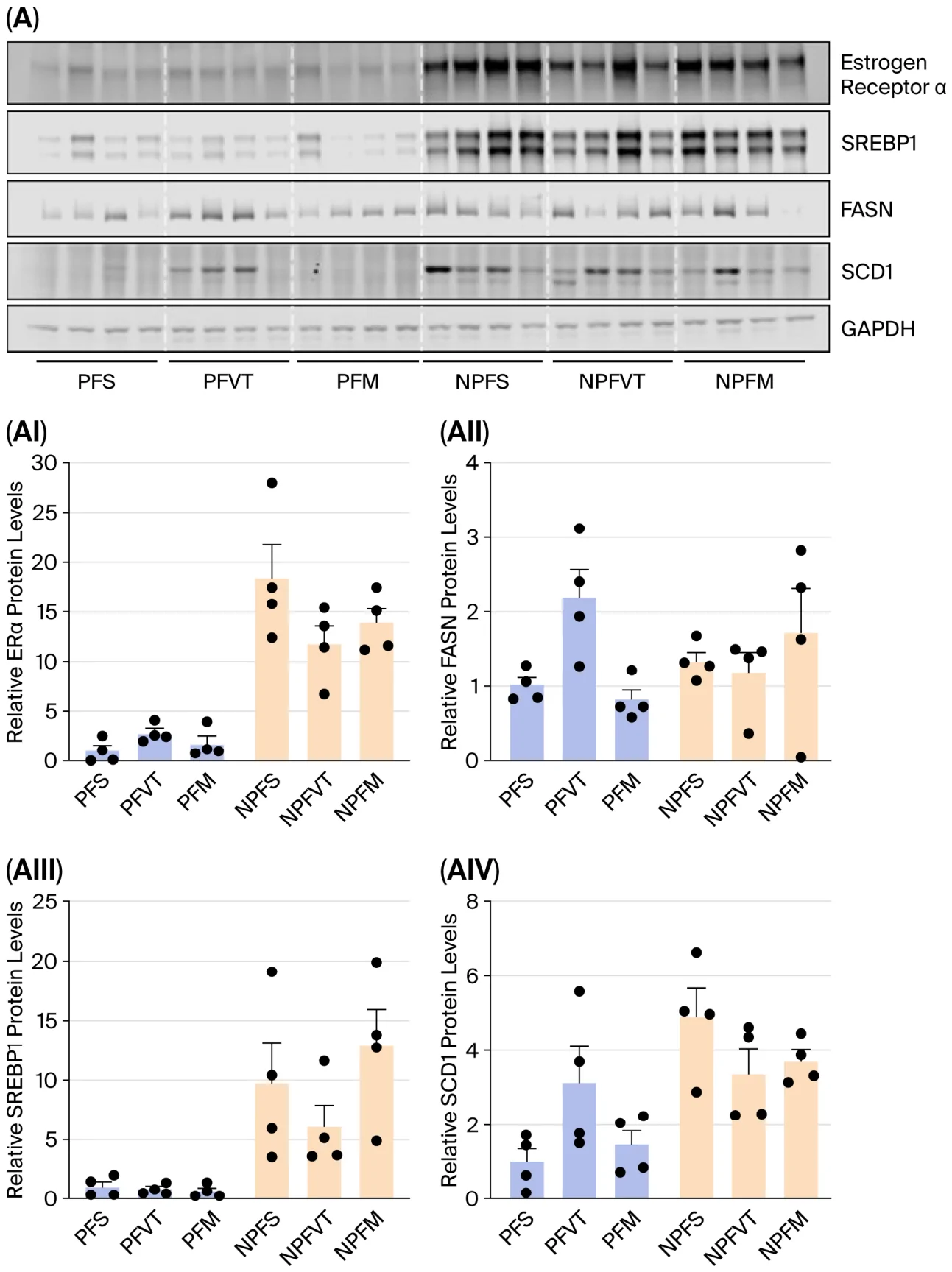
Protein expression analyses in pregnant and non-pregnant cohorts: (**A**) protein expression determined by Western blotting; (**AI**) relative Erα protein levels; (**AII**) relative *Fasn* protein levels; (**AIII**) relative *Srebp1* protein levels; and (**AIV**) relative *Scid1* protein levels. Normalization was performed using GAPDH.

Figure 6A illustrates the expression levels of key markers like SCD1, FASN, SREBP1, and estrogen receptor α. The bar graph shows quantified protein abundance, normalized to GAPDH. Figure 6AI shows the estrogen receptor-α levels in both the pregnant and non-pregnant groups. Interestingly, compared with the pregnant group, estrogen receptor-α levels were significantly higher in the non-pregnant sham group than in the non-pregnant VT and MT groups. In contrast, during pregnancy, estrogen receptor-α was slightly higher in the VT- and MT treated groups compared with the pregnant sham cohort. The SREBP1 expression level was determined via Western blotting and was found to be higher in the MT non-pregnant group, while changes in SREBP1 in the pregnant treated groups were negligible. This indicates a profound downregulation of the SREBP1 pathway during pregnancy (Figure 6AII). Figure 6AIII shows that the FASN protein level was higher in the VT-treated pregnant animal group. Interestingly, in the non-pregnant group, the FASN protein level was slightly elevated under MT exposure compared with the sham aerosol and VT. Furthermore, SCD1 was upregulated in both the pregnant and non-pregnant groups, with higher levels in the VT-treated non-pregnant group; the sham-treated group showed a slightly higher SCD1 level than the other two groups (Figure 6AIV). Hence, these findings confirm that maternal exposure to VT aerosols disrupts hepatic homeostasis by altering lipogenic genes, which are naturally suppressed in the treated pregnant liver. These findings also suggest that the metabolic risks with different flavors of vaping tobacco exposure vary, which may be further altered by the unique hormonal and immunological environment of pregnancy.

### 2.5. Exposure to Menthol Aerosol Induced Hepatic Fibrosis and Determined Organ Structural Integrity in Non-Pregnant Cohorts

**Figure 7:**
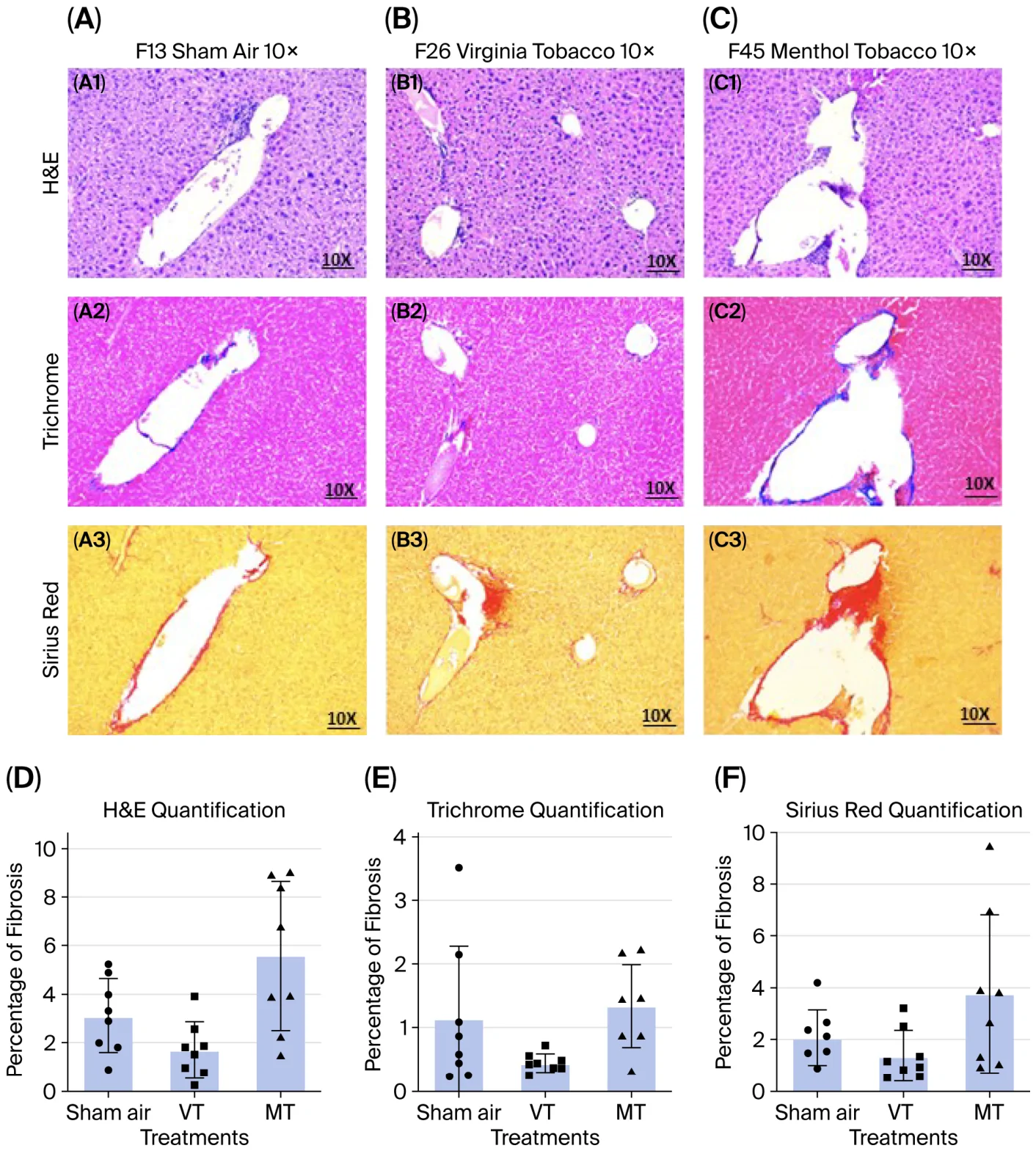
Histological study of non-pregnant cohorts. (**A**) Sham-treated liver section analysis: (**A1**) Sham H&E staining. (**A2**) Sham trichrome staining. (**A3**) Sham Sirius red staining. (**B**) Virginia-treated liver section analysis: (**B1**) Virginia H&E staining, (**B2**) Virginia Trichrome staining, and (**B3**) Virginia Sirius red staining. (**C**) Menthol-treated liver section analysis: (**C1**) Menthol H&E staining, (**C2**) Menthol trichrome staining, and (**C3**) Menthol Sirius red staining. (**D**) H&E stain quantification in all 3 treated livers; (**E**) Trichrome stain quantification in all 3 treated livers; (**F**) Sirius red stain quantification in all 3 treated livers.

To confirm the physical consequences of the observed molecular disruptions and establish a baseline toxicological profile, we conducted a targeted histopathological study in the non-pregnant cohorts. Figure 7 shows the histopathological evaluation of the non-pregnant treated group with different stains, including H&E, Trichrome, and Sirius Red. Figure 7A1–C1 show the H&E-stained qualitative analysis of liver sections from the sham, VT, and MT groups, respectively. Interestingly, the quantitative H&E results showed higher hepatic fibrosis in the MT-treated non-pregnant group than in the sham and VT groups (Figure 7D). Figure 7A2–C2 show trichrome staining results, and Figure 7A3–C3 show Sirius red staining results. Interestingly, the Menthol-treated group showed increased hepatic fibrosis, as confirmed by histological quantification (Figure 7E,F). Therefore, VT and MT exposures could possibly induce different effects in the liver. However, analysis of markers for fibrosis and inflammatory indicators should be performed to clarify the mechanism involved in the effects of these exposures.

### 2.6. VT Aerosol Exposure Initiates the Targeted Inflammatory Shift and Aggravates Immune Responses Differentially in the Livers of Pregnant and Non-Pregnant Mice

**Figure 8:**
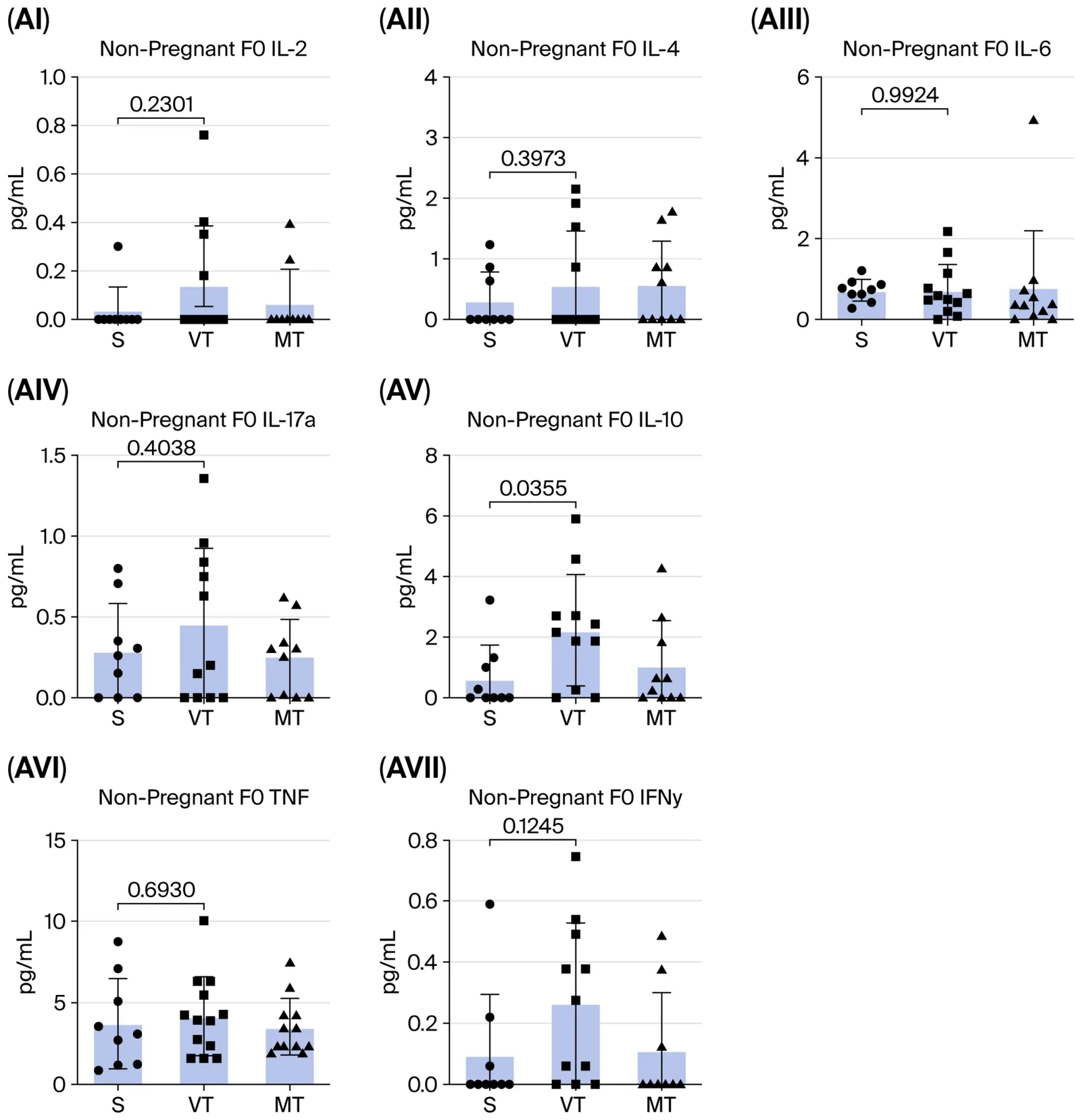
Cytokine analyses in non-pregnant cohorts. (**AI**) IL2 levels, pg/mL; (**AII**) IL4 levels, pg/mL; (**AIII**) IL6 levels, pg/mL; (**AIV**) IL17a levels, pg/mL; (**AV**) IL 10 levels, pg/mL; (**AVI**) TNF levels, pg/mL; (**AVII**) IFNy levels, pg/mL.

**Figure 9:**
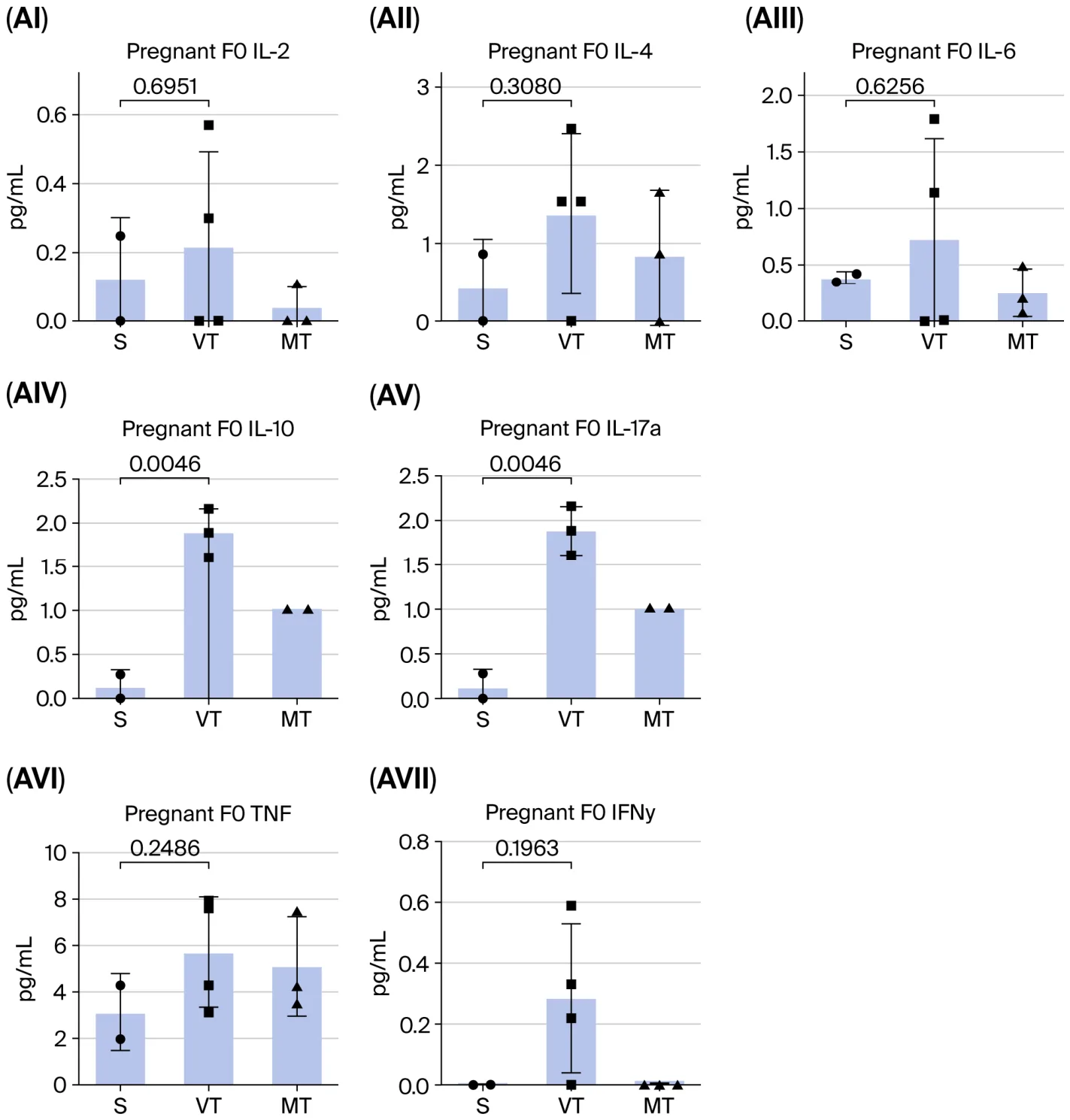
Cytokine analyses in pregnant cohorts.(**AI**) IL2 levels, pg/mL; (**AII**) IL4 levels, pg/mL; (**AIII**) IL6 levels; pg/mL; (**AIV**) IL 10 levelspg/mL; (**AV**) IL17a levels, pg/mL; (**AVI**) TNF levels, pg/mL; (**AVII**) IFNy levels, pg/mL.

To investigate the inflammation associated with VT- and MT-induced hepatic metabolic variation, we evaluated pro- and anti-inflammatory cytokines in both the pregnant and non-pregnant groups. Figure 8 illustrates the pro- and anti-inflammatory cytokine levels in the non-pregnant treated groups. Cytokines such as IL2, IL4, IL6, IL17a, IL10, TNF, and IFNy were analyzed in the sham, VT-, and MT-treated non-pregnant cohorts. Figure 8 and Figure 9 reveal that nearly all cytokines showed an increasing trend or significant rise after VT exposure in both pregnant and non-pregnant mice. Figure 8AI–AVII illustrate the cytokine levels in the non-pregnant cohort. Chronic exposure to VT led to the stimulation of anti-inflammatory cytokine IL-10 (*p* = 0.0355), with a 3-fold increase, and negligible amounts of activated pro-inflammatory IL-17a (*p* = 0.4038) as well as other markers such as TNF (*p* = 0.693), IFNy (*p* = 0.1245), IL-6 (*p* = 0.992), IL-2 (*p* = 0.230), and IL-4 (*p* = 0.3973), demonstrating the effect of VT on inflammation. Interestingly, in the pregnancy-treated cohorts, chronic VT exposure activated parallel expression of both pro-inflammatory (IL-17A, *p =* 0.0046) and anti-inflammatory (IL-10, *p =* 0.0046) cytokines (Figure 9AI–AVII). Other markers, including TNF (*p* = 0.2486), IFNy (*p* = 0.1963), IL-6 (*p* = 0.6256), IL-2 (*p* = 0.6951), and IL-4 (*p* = 0.3080), showed upward trends in the VT group; these did not reach statistical significance, indicating a targeted rather than generalized inflammatory response. This potent dual enhancement indicates that the immune conflict is unique to the maternal–tobacco interaction. These findings reveal a divergent toxicological result in which the physiological conditions during pregnancy may significantly amplify the inflammatory response to the VT aerosol.

### 2.7. Pregnancy Aggravates VT and MT Stimulation of Hepatic Mitochondrial Dysfunction and Substrate Reduction

**Figure 10:**
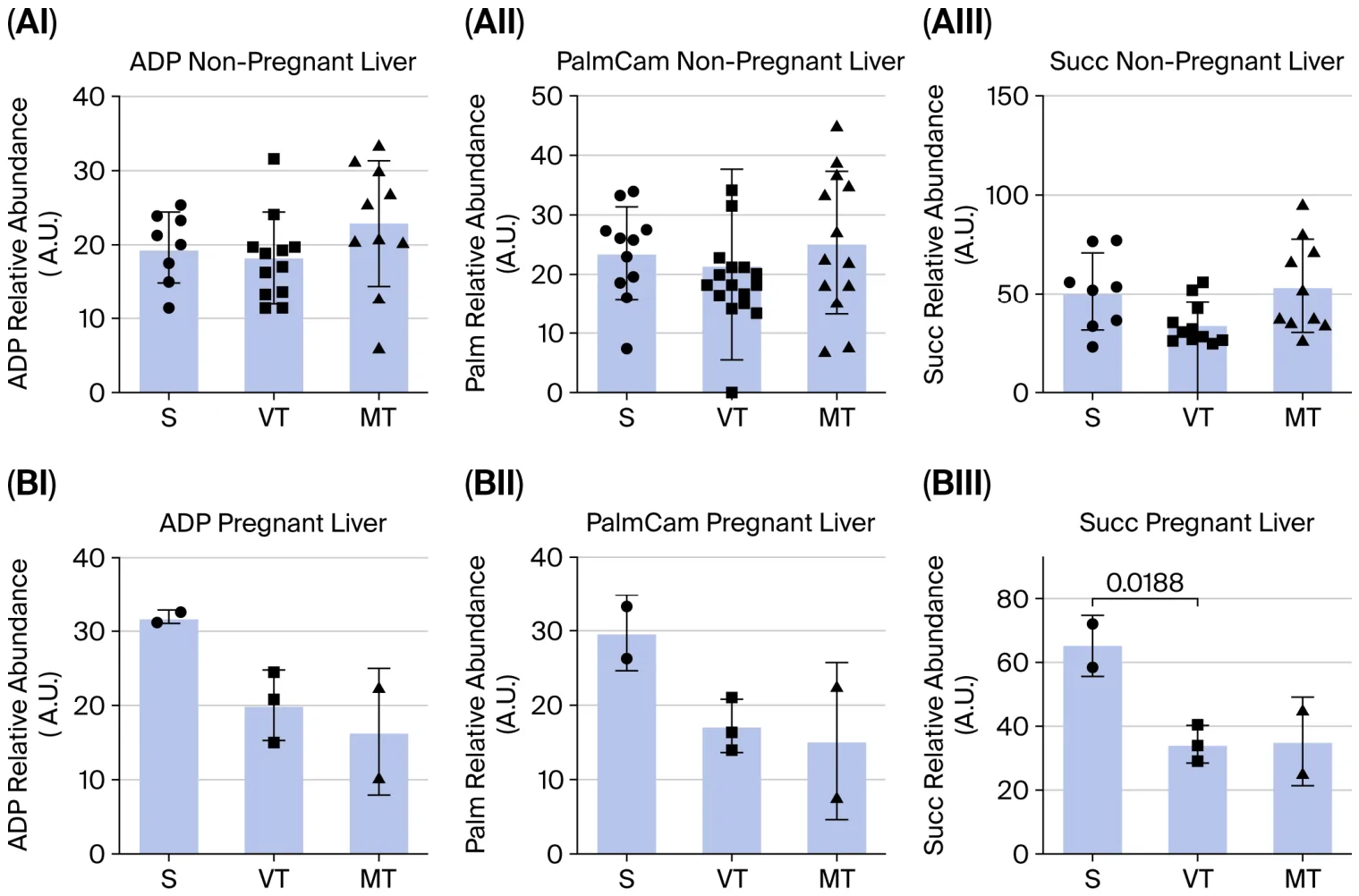
Mitochondrial functional analyses in pregnant and non-pregnant cohorts. (**A**) Mitochondrial functional analyses in non-pregnant mice: (**AI**) ADP relative abundance; (**AII**) Palm relative abundance; and (**AIII**) Succ relative abundance. (**B**) Mitochondrial functional analyses in pregnant mice: (**BI**) ADP relative abundance; (**BII**) Palm relative abundance; and (**BIII**) Succ relative abundance.

Hepatic mitochondrial oxidative function was evaluated via Oroboros O2k respirometry with substrates including glutamate/malate, ADP, palmitoylcarnitine, and succinate (Figure 10). In the livers of non-pregnant mice, we observed no significant difference in coupled respiration with glutamate/male + ADP (Figure 10AI) nor after the addition of palmitoylcarnitine (Figure 10AII) or succinate (Figure 10AIII). Interestingly, there was a slight decrease in succinate-driven respiration in the VT-treated group of non-pregnant mice. In contrast, glutamate/malate + ADP, palmitoylcarnitine, and succinate respiration were all decreased following VT and MT exposure in the livers of pregnant mice (Figure 10BI–BIII). These results suggest that vaping either VT or MT significantly reduces mitochondrial oxidative function in the livers of pregnant but not non-pregnant mice.

### 2.8. Effect of VT and MT Exposure on Hepatic Injury Markers in Livers of Non-Pregnant and Pregnant Mice

**Figure 11:**
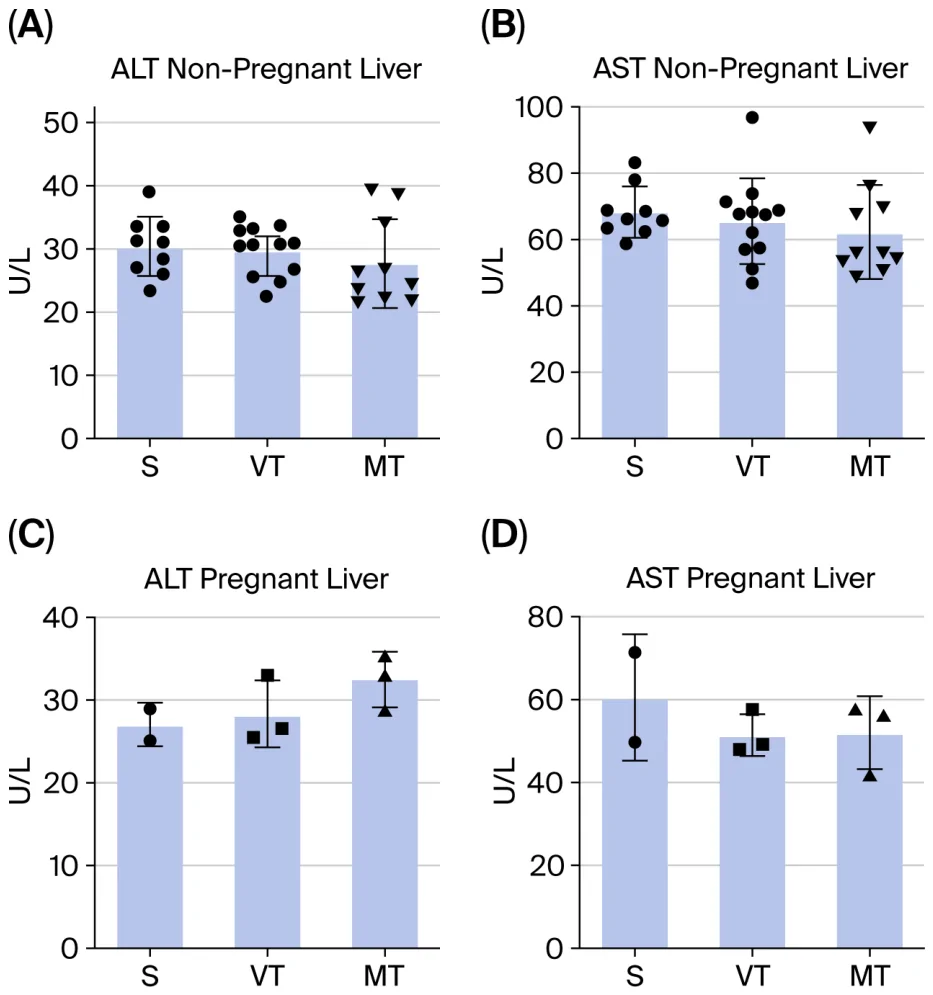
Liver enzyme analysis in pregnant and non-pregnant cohorts. (**A**) Level of ALT in non-pregnant mouse livers. (**B**) Level of AST in non-pregnant mouse livers. (**C**) Level of ALT in pregnant mouse livers. (**D**) Level of AST in pregnant mouse livers.

Hepatic injury in the livers of non-pregnant and pregnant mice was measured by analyzing serum AST and ALT levels (Figure 11). In non-pregnant mice, the liver levels of ALT and AST did not differ significantly among the sham, VT, and MT groups (Figure 11A,B). Similarly, in the pregnant groups, liver ALT levels showed a modest, non-significant upward trend across the sham, VT, and MT groups (Figure 11C). AST levels were comparable across all three groups, with no statistically significant differences. Collectively, these results indicate that neither VT nor MT exposure significantly altered markers of hepatocellular injury (ALT, AST) in non-pregnant and pregnant mice, suggesting that the doses and durations of eCig exposure used in this study may have been the primary causes of pulmonary damage and inflammation.

## 3. Discussion

This study presents new insights into the pathophysiological impact of chronic exposure to vaping products with different flavor constituents and their distinctive pathophysiological nature during pregnancy [8]. Vaping products have been considered safer alternatives to traditional combustible cigarettes, a belief that likely originates from these products initially being marketed as smoking cessation aids and nicotine-free products [9]. However, this belief has been challenged following reports of side effects of vaping on various organ systems (e.g., digestive, respiratory, cardiovascular). Currently, there is no conclusive scientific research proving that it is safe to use e-cigarettes during pregnancy, and the nicotine in e-cigarettes can still have some negative effects in other forms of delivery [10]. The findings in this study may have clinical relevance, as 19–32% of adults of child-bearing age have reported using eCigs at least once in their lifetime, and 4–10% are current daily users. While eCig use is rapidly increasing among teens, only a small percentage of adult women of reproductive age are current daily users. Nevertheless, as there is no reliable proof of the safety of e-cigarettes for expectant mothers and their babies, it is recommended that they not be used during pregnancy.

The hormonal, immunologic, and lipid metabolic changes that occur during pregnancy to support fetal growth and development may render an animal more vulnerable to the detrimental effects of environmental exposure. Data from this study demonstrate that chronic exposure to VT and MT aerosols resulted in differential biological responses, including pulmonary inflammation, depending on whether the animals were pregnant or not. VT exposure led to pulmonary inflammation during pregnancy, while MT exposure led to more pulmonary inflammation in the non-pregnant state. These data suggest that VT and MT aerosols induced pulmonary inflammation through different mechanisms. These differential responses might be due to different flavoring chemicals and chemicals formed in the aerosol that affect oxidative stress, epithelial damage, and inflammatory signaling [11]. However, without directly evaluating the individual constituents and their contributions, the mechanisms of the different responses to VT and MT remain undetermined. Relatively little is known about how physiological and immunological changes during pregnancy influence responsiveness to inhaled aerosols. The lack of a pulmonary inflammatory response to menthol during pregnancy may be compensatory and protective for fetal development.

This study shows that vaping disturbs the liver metabolic equilibrium during pregnancy. This is a critical finding, as there was evidence of derangement of lipogenic gene expression in the livers of the non-pregnant and pregnant mice. Maternal exposure to VT aerosols disrupted hepatic homeostasis by increasing lipogenic gene expression, as Scd1 and Fasn levels were elevated in these mice. In the third trimester of pregnancy, these genes are suppressed to allow more circulating fatty acids to provide nutrients, glucose, and ketones to the fetus. While most lipogenic gene changes were observed in the VT-exposed mice compared with the sham, the MT-treated cohorts showed the highest fold difference in liver triglycerides. In addition, the MT-exposed mice showed evidence of heightened liver fibrosis. These findings suggest that there are metabolic risks associated with exposure to different flavors of vape tobacco. These metabolic risks may be further altered by the unique hormonal and immunological environment of pregnancy. While nicotine may contribute to the lipogenic changes associated with VT exposure, other constituents found that in eCig-VT exposure, hormonally mediated mechanisms may also be involved, raising new health concerns regarding these products’ constituents and flavoring.

The findings of this study also highlight disturbances in the systemic inflammatory equilibrium during pregnancy. Hepatic differences in response to VT and MT exposure may be due to pregnancy-associated pharmacokinetic differences. Inhaled constituents can cross the pulmonary epithelium, enter systemic circulation, and reach the liver. Respiratory physiology changes and drug-metabolizing enzyme activity during pregnancy may affect internal exposure to the constituents and metabolites of inhaled drugs [1,2,3,4]. Nicotine is primarily metabolized by hepatic CYP2A6, while menthol is metabolized primarily by glucuronidation and oxidation [12,13]. It is possible that the observed variations in hepatic lipid metabolism, fibrosis, and mitochondrial function are due to changes in toxicant exposure and/or tissue susceptibility due to pregnancy. However, the concentrations of these toxicants within body tissues were not measured in this study, so further research is necessary to separate pharmacokinetic from toxicodynamic effects. It is recognized that pregnancy results in a state of immune balance and tolerance to facilitate the growth of the fetus. During pregnancy, exposure to both VT and MT aerosols led to increases in both IL-17A and IL-10 compared with sham-treatment exposure. In the non-pregnant state, only IL-10 was activated following VT exposure, although there was a trend of increase for all cytokines after VT exposure. IL-17 may be pro-inflammatory at times, causing tissue damage; however, IL-10 is anti-inflammatory, which counteracts this damaging function. However, IL-17 has also long been considered to have an anti-inflammatory effect depending on the dose. Although the mechanism is still unclear, the results suggest that pregnancy may trigger a complex immune reprogramming process to tolerate exposure to VT and MT. However, key questions remain.

The global response to these exposures involves molecular, histopathological, and immunological changes. Factors that may play a role include how the different flavors and e-Cig ingredients trigger potential toxicity by activating the inflammasome, inducing ferroptosis, altering mitochondrial biology, or even changes in the epigenome [14]. The potential inflammatory response to e-Cig exposure might involve activation of the NLRP3 inflammasome, which may further promote the release of inflammatory cytokines such as IL-1β and IL-18 by processing caspase-1 [15]. Although IL-1β, IL-18, and caspase-1 were not measured in this study, there are published data to suggest that IL-10 activation may occur to regulate NLRP3 inflammasome activation. Moreover, with VT and MT exposure, the special role of the placenta in maternal–fetal interactions cannot be underestimated.

This study has several limitations, as these findings are more suggestive of potential associations rather than causality regarding the metabolic and immunologic responses to VT and MT during pregnancy. More studies are needed to determine if these findings are reproducible in human patients. As many of the biomarkers of health and disease with respect to vaping have not been identified, more attention should be paid to maternal and placental signaling, oxidative stress response, and fetal outcomes that may be influenced by vaping prior to or during pregnancy. However, although not yet proven in human patients, these findings suggest that the ingredients in VT and MT aerosols interact with the metabolic and immunologic adaptations of gestation to induce diverse inflammatory effects (more specific to VT-exposed mice), liver fibrosis responses (specific to MT-exposed mice), lipid accumulation (in both groups but to different extents and causes), or functional mitochondrial impairment (in both VT- and MT-exposed pregnant mice). Thus, this study represents a bridge in research on the impact of gestation on tobacco toxicology and points toward the complex interrelationship between gestation, vaping exposure, and risks to maternal and offspring well-being.

One persistent concern with these exposures is whether the biological and biochemical changes can be transmitted to offspring or subsequent generations via alterations in DNA methylation, histone modifications, or changes in miRNA expression [16]. One point of caution with the difference in biological profile between VT and MT is that although liver lipidomic genes are disrupted in pregnancy, mostly due to VT, there is a uniform reduction in liver tissue mitochondrial function related to both VT and MT vape use during pregnancy. Mitochondrial abnormalities account for fat accumulation and tissue damage [17]. Substrates like ADP (related to ATP hydrolysis), palmitoyl carnitine (involved in fatty acid entry into the mitochondria), and succinate (a Krebs cycle enzyme) are all essential regulators of ATP synthesis, substrate influx, and fatty acid oxidation and are directly indicative of how mitochondrial respiration and substrate utilization capacity could be impaired by exposure to VT or MT products. Impaired mitochondrial respiration may lead to excessive ROS generation, lipid deposition, and ultimately triggers inflammation and tissue or cellular structural changes that may have a health impact. The differential hepatic responses between VT and MT exposure may also be driven by differences in the composition of the products tested. Prior studies have reported differences in the chemical composition of JUUL products and have proposed that the chemical chemicals associated with the flavors may contribute to differences in cellular toxicity and mitochondrial dysfunction [18,19]. While we matched the puff-topography parameters, we did not measure the chemical composition, nicotine yield, or yield of the aerosols generated from the two products; thus, we cannot conclude equivalence in chemical exposure. Thus, our findings should be interpreted as a comparison of the two tested commercial products, not as a demonstration of flavor effects in isolation. The specific constituents responsible for the differences in pulmonary inflammation, hepatic responses, and mitochondrial function are unknown. Additional studies including chemical and aerosol characterization and mechanistic experiments are needed to delineate the possible effects of VTT and MTT on the different biological endpoints studied and the contribution of pregnancy.

## 4. Materials and Methods

### 4.1. Chemical Reagents

JUUL(JUUL Labs, San Francisco, CA) Virginia tobacco (VT), Menthol (MT), total protein kit, LDH kit, chloroform, methanol Pierce BCA Protein Assay Kit (Thermo Cat#23227, Rockford, IL, USA), RNeasy plus kit (Qiagen Cat#73404, Germany), TaqMan Reverse Transcription Reagents kit (catalog number N8080234; Applied Biosystems, USA) 5% skimmed milk, Tween-20, 10% SDS, Acrylamide, Tris-Hcl buffer, TEMED, Ammonium persulfate, GAPDH (Cell Signaling Cat#2118, Danvers, MA, USA), Horse radish peroxides, sterol regulatory element-binding protein 1 (SREBP1, Cell Signaling Cat# 95879, Danvers, MA, USA), fatty acid synthase (FASN, Cell Signaling Cat#3189, Danvers, MA, USA), stearoyl-CoA desaturase-1 (SCD1, Cell Signaling Cat#2794, Danvers, MA, USA), and estrogen receptor alpha (Erα Cell Signaling Cat#13826, Danvers, MA, USA).

### 4.2. Institutional Approval: Animal

#### Animal Grouping

All animal procedures were approved by the Saint Louis University IACUC Protocol 3089 and performed in strict compliance with the institution’s guidelines on laboratory animal care and use. Female C57BL/6J mice were chosen because their physiology, including metabolism and reproduction, is well-characterized and widely utilized in inhalation toxicology. The mice were maintained in a 12 h light–12 h dark environment with the temperature controlled at 22 ± 2 °C and the humidity maintained at 40–60%. The mice were provided ad libitum access to commercial rodent chow and water.

After a 1-week acclimatization period, female mice were randomly allocated to one of the following treatment groups: (1) sham-exposed animals (control); (2) animals exposed to Virginia tobacco aerosol (VT); and (3) animals exposed to Menthol aerosol (MT). This approach was designed to test whether differences in the components of tobacco aerosols or animal physiological state (pregnant vs. non-pregnant) lead to disparate responses in lung inflammation, liver lipid metabolism, mitochondrial function, and systemic inflammation. A murine model was used to investigate the health effects of vaping aerosols and their potential roles in chronic illnesses.

### 4.3. Exposure Method

Female mice were exposed for 6 weeks prior to mating with e-Cig-naive males. The control and sham groups were exposed to filtered room air alone. Exposures occurred over 1 h daily for 5 days, with a puffing regime consisting of 4 s puff and 16 s inter-puff intervals. This exposure yielded 3 puffs/min at a 2LPM total airflow and a final puff volume of ~36 mL. Additionally, body weights and physiological assessments were analyzed in the grouped animals. Body weight was recorded weekly for up to 13 weeks, and physiological changes were also noted for both the pregnant and non-pregnant cohorts. After 13 weeks, the animals were anesthetized, tissue and blood samples were collected using a standard protocol, and the collected samples were used for further analysis.

### 4.4. Pulmonary Inflammation Analysis

Pulmonary inflammation was determined through bronchoalveolar lavage fluid analysis like previously performed method [20]. Following euthanasia, the trachea was gently lavaged three times while being incubated with a sterile catheter filled with phosphate-buffered saline (PBS). The recovered lavage fluid was pooled and centrifuged at 400× g for 10 min at 4 °C. The supernatant was collected for biochemical analyses, and the cell pellet was used to determine total BAL cell counts. Cell viability and total viable cell numbers were assessed using trypan blue exclusion and a hemocytometer. This method quantified total viable BAL cells but did not distinguish individual inflammatory cell populations. The concentration of BAL was represented as total BAL cells, total cells per mouse, and % of cellular recruitment. Additionally, the total protein concentration (Cat. No. 23227, Rockford, IL, USA) and lactate dehydrogenase (LDH) activity were measured (Cat log NO C20300,) lot 3436129, Invitrogen, Eugene, OR, USA) in the BAL fluid residue using the manufacturer’s instructions.

### 4.5. Quantification of Triglycerides

Triglyceride levels were evaluated using liver tissues from both the pregnant and non-pregnant mice. The collected liver tissues were weighed for the required amount and homogenized, and lipids were extracted using a chloroform–methanol extraction procedure based on the Folch method [21]. Triglycerides were then measured with a colorimetric assay kit ((Cat log NO # ETGA-200) lot CB06A24,Bio assay System, USA) according to the manufacturer’s instructions.

### 4.6. Analysis of Hepatic mRNA Expression

#### Isolation of RNA and cDNA Synthesis

Total RNA was extracted from the liver tissues of both pregnant and non-pregnant mice according to a previous protocol [22] using a RNeasy Plus kit (QIAGEN, catalog number 73404, Germany). Quantification and quality control of the extracted RNAs were performed using a Nano-drop spectrophotometer and RNA Nano-drop with an Agilent 2100 bioanalyzer (Agilent Technologies), respectively. Reverse transcription of 1 µg of total RNA was performed in a 20 µL reaction mixture with a Reverse Transcription Reagents kit from ABI (catalog number N8080234; Applied Biosystems, Foster City, CA, USA). Amplification with qPCR was performed in triplicate and consisted of 1 µL of cDNA in a total volume of 25 µL of Fast Start Universal SYBR Green Master Mix (Roche Applied Science #04913850001, IN, USA). mRNA sequences of lipogenic regulators and downstream enzymes included SCD1 (Stearoyl-CoA desaturase-1), FASN (Fatty acid synthase) and SREBP1 (Sterol regulatory element-binding protein-1). These were selected as targets to describe the maternal metabolic bypass in response to Virginia tobacco. GAPDH was used as an endogenous housekeeping control for all the reactions. PCR assays were performed using ABI Prism 7500 Sequence Detection System. Triplicate cycle threshold (CT) values were analyzed using the comparative CT (ΔΔCT) method, according to the manufacturer’s instructions (Applied Biosystems, IN, USA). The amount of target (2-ΔΔCT) was obtained by normalization to the endogenous reference (18S) sample. RNA isolation, primer design, and RT-qPCR were performed using Molecular Genomics Core, UTMB, Galveston, TX, USA.

### 4.7. Western Blot Analysis of Hepatic Lipid Metabolism-Related Proteins

Liver tissues were lysed with RIPA lysis buffer. The total protein content of each sample was detected using the BCA Protein Assay Kit (Pierce, Rockford, IL, USA). Protein separation by 10% precast SDS-PAGE polyacrylamide gels (BioRad Lab, Hercules, CA, USA) and transferred onto a nitrocellulose membrane (BioRad Lab) was performed according to the standard WB protocol as described previously [23]. Membranes were blocked in 5% skimmed milk with Tween-20 (0.1%) at room temperature for 1hr to minimize non-specific antibody binding. These membranes were then incubated with the respective primary antibodies (sterol regulatory element-binding protein 1 (SREBP1), fatty acid synthase (FASN), stearoyl-CoA desaturase-1 (SCD1), and estrogen receptor alpha (ER)) overnight at 4 °C. Subsequent washes were performed, and the membranes were then incubated with appropriate secondary antibodies for 2 hr at room temperature to detect the respective protein bands, which was facilitated by a chemiluminescence detection reagent.

### 4.8. Histopathological Analysis for Non-Pregnant Mouse Liver Tissue

Histopathological examination was performed as previously described [24]. After the animals were euthanized, liver samples were collected and fixed in 10% formalin solution for 24–48 h. The samples were dehydrated using a series of ethanol grades and then cleared with xylene and embedded in paraffin blocks. The samples were sectioned using a microtome to a thickness of 4–5 μm, fixed in slides, and stained using H & E (hematoxylin and eosin), Mason’s trichrome, and Sirius red. Subsequently, structural modifications in the liver samples were recorded and photographed under a light microscope.

### 4.9. Analysis of Cytokines

Pro-inflammatory (IL-2, IL-6, IL-17A, TNF-α, and IFN-γ) and anti-inflammatory cytokines (IL-10 and IL-4) were measured in serum samples from both the pregnant and non-pregnant groups. Cytokine measurements were performed using a multiplex bead-based immunoassay platform (Luminex) according to a published protocol [25] and the manufacturer’s guide. The serum samples and standards were incubated with coated magnetic beads, which attracted and captured the targeted molecules from the sample. After the detection of antibodies, a fluorescence signal was produced and quantified using Luminex200 (Bio-rad Laboratories, Hercules, CA, USA). Data are expressed in pg/mL.

### 4.10. Analysis of Hepatic Mitochondrial Function

Hepatic mitochondrial respiration was analyzed in 1–3 mg of fresh liver tissue sliced with a razor blade and permeabilized with 5 μg/mL digitonin in the 2 mL respiration chambers of an Oxygraph O_2_k FluoRespirometer (Oroboros Instruments, Innsbruck, Austria). Mir05 respiration buffer was used and prewarmed to 37 °C within the chambers. Prior to sealing the chambers, ~1 mL of 100% oxygen was injected into the partially closed chambers to raise the soluble oxygen levels to ~400 μM so that oxygen diffusion into the tissue chunk was not limited. After sealing the chambers and stabilization of the oxygen levels, substrates were sequentially added using a Hamilton syringe, starting at 10 min post digitonin permeabilization. After each substrate addition, oxygen consumption rates were allowed to come to a steady state before the next addition. The substrates were added in the following order: 10 mM glutamate + 2 mM malate, 2.5 mM ADP, 0.5 mM palmitoyl carnitine, and 10 mM succinate. Data for oxygen flux or oxygen consumption rate are expressed as pmol O_2_/s/mg tissue [26].

### 4.11. Analysis of Hepatic Enzymes

The effect of tobacco aerosols on the activity of serum alanine transferase (ALT) and aspartate aminotransferase (AST) was assayed using previously described methods [27]. After necropsy, blood samples were collected, serum was separated, and levels of liver enzymes, serum alanine transferase (ALT), and aspartate aminotransferase (AST) were tested using commercial assays.

### 4.12. Statistical Analysis

Grouped results are expressed as the mean ± SD. The data were normalized via natural log transformations and, when normally distributed, statistical significance among the groups was determined using one-way ANOVA with Bonferroni correction with multiple pairwise comparisons. When the data were not normally distributed, we used the Kruskal–Wallis assessment of ranks followed by Dunn’s multiple comparison tests. Student’s *t*-test was employed for comparison between two groups with a 95% confidence interval using Prism software-8 (GraphPad Software Inc., San Diego, CA, USA). A *p* ≤ 0.05 was considered statistically significant. Real-time PCR results were analyzed by normalizing the data first to the housekeeping gene and then to the average of each control group (lung/heart).

## 5. Conclusions

The present study compared the effects of vaping between pregnant and non-pregnant female mice cohorts exposed to Virginia tobacco and Menthol electronic cigarettes, along with control sham-treated mice. This study was conducted over 13 weeks and primarily compared the health implications of vape exposure between the pregnant and non-pregnant groups. The study showed that exposure to VT aerosols during pregnancy altered hepatic lipogenic genes. Furthermore, in non-pregnant mice, VT caused more pronounced cytokine abnormalities, and histological staining indicated worsening liver fibrosis in the MT-exposed mice. Both VT- and MT-exposed mice showed reduced mitochondrial liver function during pregnancy. More importantly, this study highlights the complex milieu of immune reprogramming that may occur in pregnant mice during pregnancy to tolerate vaping exposure, with less evidence of overt inflammation. The health implications and translational values of these findings are unclear, as most findings are subclinical. More research is needed to assess if these results can be replicated in human patients and to elucidate the mechanisms linking vaping exposure to potential long-term hepatic injury.

## Data Availability

The original contributions presented in this study are included in the article. Further inquiries can be directed to the corresponding authors.

## Abbreviations

The following abbreviations are used in this manuscript:

eCigs—electronic cigarettes, ENDS—electronic nicotine-delivery systems; VT—Virginia tobacco; MT— Menthol; BALF—bronchoalveolar lavage fluid; LDH—lactate dehydrogenase; SCD1—stearoyl-CoA desaturase-1; FASN —fatty acid synthase; SREBP1—sterol regulatory element-binding protein—1; GAPDH—glyceraldehyde 3-phosphate dehydrogenase; IL—interleukin; TNF—tumor necrosis factor; INF— interferon; SREBP1—sterol regulatory element-binding protein 1; FASN—fatty acid synthase; SCD1—stearoyl-CoA desaturase-1; ERα—estrogen receptor alpha; ALT—alanine aminotransferase; AST—aspartate aminotransferase; ADP—adenosine diphosphate.
